# Supraclavicular metastasis from prostate cancer: unusual case and literature review

**DOI:** 10.1093/jscr/rjad091

**Published:** 2023-03-07

**Authors:** Noelani-Mei Ascio, Abid Qureshi, Elliot Banayan, Armand Asarian, Romulo Genato, Philip Xiao

**Affiliations:** School of Medicine, St. George’s University, Grenada, WI, USA; Department of Surgery, The Brooklyn Hospital Center, Brooklyn, NY, USA; Department of Surgery, The Brooklyn Hospital Center, Brooklyn, NY, USA; Department of Surgery, The Brooklyn Hospital Center, Brooklyn, NY, USA; Department of Surgery, The Brooklyn Hospital Center, Brooklyn, NY, USA; Department of Pathology, The Brooklyn Hospital Center, Brooklyn, NY, USA

## Abstract

Prostate cancer is the most common cancer among men, with the most common metastatic sites in bone, regional lymph nodes, liver and thorax. It is most commonly diagnosed in the early stages with clinical findings of enlarged prostate on digital rectal exam and positive prostate specific antigen. Distant metastases associated with prostate cancer commonly occur to bone. It is imperative to be cautious in assuming primary breast, lung or head and neck malignancy in patients presenting with lymphadenopathy in the upper aerodigestive pathways. Cervical lymphadenopathy due to prostate cancer is becoming more prevalent since previously reported. Here we present a case of prostate cancer recurrence found through metastasis to supraclavicular lymph nodes and we also highlight homeobox protein CDX2 as a potential clinico-pathological marker in metastatic prostate cancer.

## INTRODUCTION

Prostate cancer is the most common cancer among men, with the most common metastatic sites in bone, regional lymph nodes, liver and thorax [1]. According to the Surveillance, Epidemiology and End Results Program, the incidence of prostate cancer between 2010 and 2019 for localized cases (‘confined to organ of origin’) was 71.4%, regional cases (‘the area extending from the periphery of an involved organ’) being 12.7% and distant metastatic cases (a tumor that has spread remotely from the primary tumor) were found in 6.8% [2]. Previously, there have been cases of supraclavicular lymphadenopathy as a site of metastasis from prostate cancer but it is an uncommon occurrence. The current literature on metastatic pathways in prostate cancer is not well understood as to whether prostate cancer spreads hematogenously or lymphatically. Here we present a case of prostate cancer recurrence found through metastasis to supraclavicular lymph nodes and we also highlight homeobox protein CDX2 as a potential clinico-pathological marker in metastatic prostate cancer.

## CASE PRESENTATION

A 75-year-old male came in with initial complaints of a left-sided neck lump for 2 months. He had associated fatigue and weight loss of 30 pounds over the last 5 years. He denied any associated fevers, chills, night sweats, pain, cough, shortness of breath, dysphagia or urinary symptoms. The patient was a chronic smoker and had a past medical history of prostate cancer status post focal radiation therapy ⁓5 years ago. He was in remission from that time period. Physical examination revealed non-tender and mobile cervical lymphadenopathy, 4 cm in size. Digital rectal examination was negative for prostatic enlargement. The chest X-ray was unremarkable. Computed tomography scan of the neck showed several enlarged lymphadenopathies at the base of the left neck/left cervical region with formation of a mass of uncertain etiology. The patient was subsequently referred to the oncology department and sent for biopsy. Biopsy of the left cervical lymph node revealed fragments of adenocarcinoma and tumor cells were positive for prostatic specific acid phosphatase (PSAP), Alpha mehyacyl CoA racemase (AMACR) and CDX2. Patient was non-compliant with medical recommendations following the operation and was lost to follow up for any further workup.

## PATHOLOGIC FINDINGS

**Figure 1:**
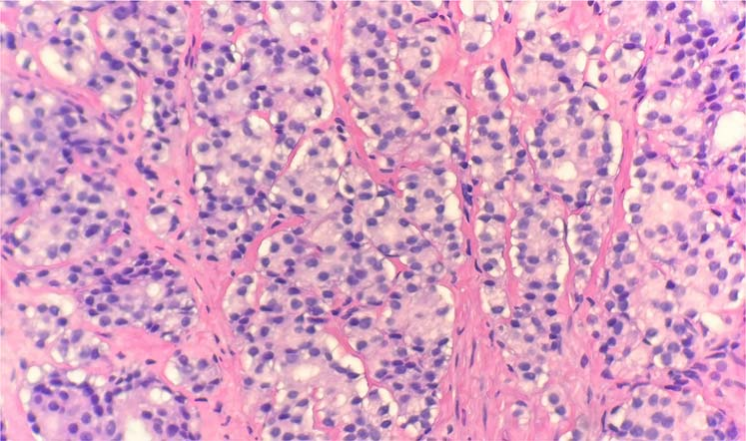
Microscopic examination reveals sheets of tumor cells with invasive growth pattern (H&E stain 40×).

**Figure 2:**
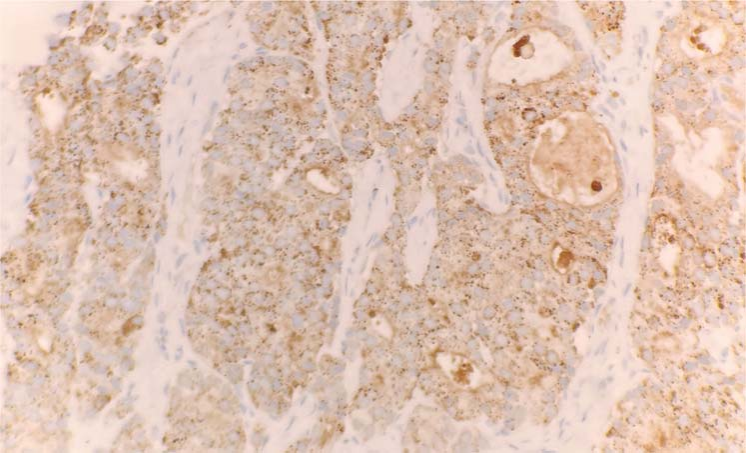
Tumor cells expressed PSAP on immunohistochemical stain (IHC stain 40×).

**Figure 3:**
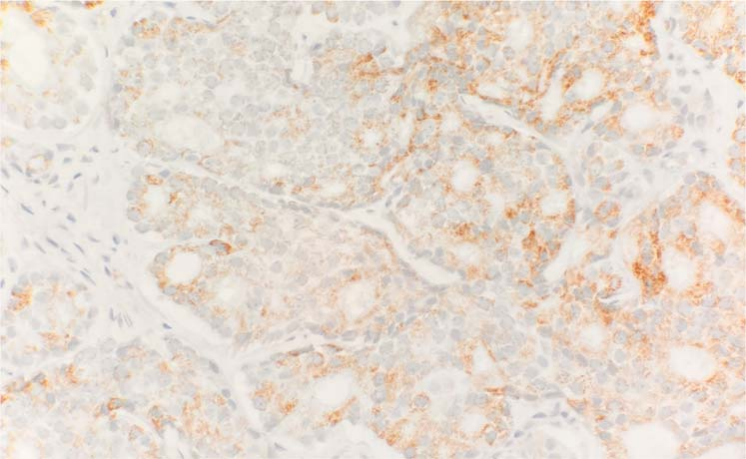
Tumor cells are positive for Alpha methyacyl CoA racemase (p504s) (IHC stain 40×).

**Figure 4:**
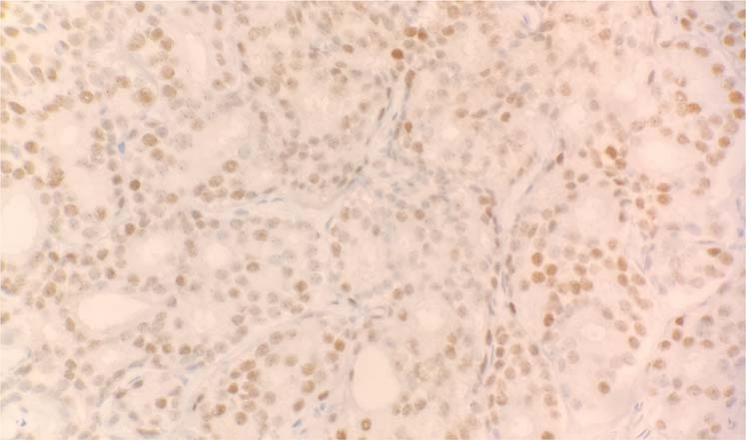
Tumor cells expressed CDX2 on immunohistochemical stain (IHC stain 40×).

Specimen received in formalin and consists of multiple needle cores soft tissue measuring up to 1.2 × 0.1 cm. Microscopic examination reveals sheet of tumor cells forming nest or trabecular invasive pattern (Fig. 1). Tumor cell are immunopositive for PSAP (Fig. 2) and Alpha methyacyl CoA racemase (p504s) (Fig. 3), and focally positive for CDX2 (Fig. 4). Negative staining for CK7, TTF1 and Napsin A make lung primary less likely. Negative staining for CK20 speaks against lower GI tract primary. Combined with morphological features, this immunoprofile supports the diagnosis of metastatic adenocarcinoma with patient’s known history of prostate primary.

## DISCUSSION

Prostate cancer is most commonly diagnosed in the early stages with clinical findings of enlarged prostate on digital rectal exam and positive prostate specific antigen (PSA). Distant metastases in association with prostate cancer commonly occur to bone but the findings of atypical metastasis should not be overlooked. Cervical lymphadenopathy due to prostate cancer is becoming more prevalent since previously a reported 0.4% incidence in 2017 [3]. In a retrospective review of cytopathology files, 13 cases were identified involving cervical lymph node metastasis, 12 of which had a known history of prostate adenocarcinoma and one that was initially misinterpreted as papillary thyroid carcinoma [4]. It is imperative to be cautious in assuming primary breast, lung or head and neck malignancy in patients presenting with constitutive symptoms with lymphadenopathy in the upper aerodigestive pathways.

It is most widely hypothesized that prostate cancer metastases occur through hematogenous spread via Batson’s plexus of lymph nodes draining from the prostate to spine. A study previously conducted from 1967 to 1995 showed the presence of hematogenous metastasis in 35% of all patients with prostate cancer occurring most commonly in bone (90%), lung (46%), liver (25%), pleura (21%) and adrenals (13%) [5]. However, this does not explain metastases spreading to left cervical lymph nodes as it would likely occur as bilateral lymphadenopathy [6]. A case series of 309 patients presenting with supraclavicular masses were biopsied, of which 55% were metastatic (47% from lung, breast or cervix, and 8% from lymphoma) concluding that cervical lymphadenopathy commonly occurring due to the primary sites listed in the study [7]. It has also been postulated that the majority of left cervical lymphadenopathy for tumor metastases occurs from the left thoracic duct to the left subclavian vein [8].

Despite the uncertain guidelines of prostate cancer screening, PSA is a routinely used marker for initial assessment and follow up, other markers used include prostein P501s, NKX3.1 and PSMA [9]. Immunoreactive markers reported most frequently found in adenocarcinoma of the prostate are NKX3.1 (99%), PSMA (92%) and PSA (88%) previously seen in a study focusing on clinico-pathological correlations of prostate metastasis [10]. CDX2 is a transcription factor protein expressed in epithelial cells of the intestine studied previously in the formation of intestine and anus in the fetal period. Interestingly, CDX2 is identified as a metastatic marker closely associated with colorectal carcinoma. It is highly uncommon to find a metastatic marker of intestinal origin in prostate cancer. There have been rare reports of prostate adenocarcinoma in association with strongly positive CDX2 metastasis as well as other reports being weakly positive [11]. CDX2, although primarily an intestinal marker, may be a possible diagnostic value in evaluating for metastasis in carcinomas of unknown origin. Being that it has been found in different cancers, it may clue into the way cancer is metastasizing if studied further.

The case presented in our report had cervical lymphadenopathy, absence of urinary symptoms and non-enlarged prostate, findings non-suggestive of prostate cancer. Prior history of radiated prostate cancer in remission and subsequent immunohistochemical findings of PSAP and AMACR on cytopathology were all cofactors to signal metastasis due to prostate cancer. Previously, a similar case has been documented of a patient without urinary tract symptoms and cervical lymphadenopathy and was found to have prostatic adenocarcinoma as well, who was treated with androgen inhibitors with regression of lymph node sizes; although CDX2 was not positive in this case [3].

## CONCLUSION

Adenocarcinoma of the prostate has an indolent course, which may be difficult to diagnose in its early stages. Cervical lymphadenopathy seems to be an atypical association with prostate cancer but it may be more prevalent than previously reported. Due to both prostate and colon cancer being the most common types of malignancies in the USA, it is important to differentiate metastases of unknown origins with immunohistochemical staining for determining primary malignancy as that will direct the course of treatment and prognosis. The rarity of CDX2 found in this case of prostate cancer metastasis to supraclavicular lymphadenopathy may add to the delayed diagnosis of the primary tumor origin. This case highlights the need to keep prostate cancer as a differential diagnosis when metastases of unknown origin present in supraclavicular lymphadenopathy and to further investigate the role of CDX2 as a biomarker in prostate cancer metastasis.

## CONFLICT OF INTEREST STATEMENT

None declared.

## FUNDING

None.
